# A Giant Stent for Giant Cerebral Aneurysms—The Accero^®^-Rex-Stent

**DOI:** 10.3390/jcm13020388

**Published:** 2024-01-10

**Authors:** Hermann Kraehling, Burak Han Akkurt, Mohamed Elsharkawy, Ahmed Ayad, Mostafa Ergawy, Ekin Celik, René Chapot, Wolfram Schwindt, Christian Paul Stracke

**Affiliations:** 1 University Clinic for Radiology, University of Münster, University Hospital Münster, Albert-Schweitzer-Campus 1, 48149 Münster, Germany; 2 University Clinic for Radiology, Department for Interventional Neuroradiology, University of Münster, University Hospital Münster, Albert-Schweitzer-Campus 1, 48149 Münster, Germany; 3 Department of Neuroradiology, Alfried-Krupp-Krankenhaus, Alfried-Krupp-Straße 21, 45131 Essen, Germanyrchapot@icloud.com (R.C.); 4 Department of Radiology and Neuroradiology, Ludmillenstift Hospital, Ludmillenstrasse 4-6, 49716 Meppen, Germany; 5 Clinic and Policlinic for Diagnostic and Interventional Neuroradiology, University Hospital Hamburg Eppendorf, Martinistraße 52, 20246 Hamburg, Germany

**Keywords:** giant intracranial aneurysm, endovascular treatment, neuroradiological intervention, intracranial stenting, coated stents

## Abstract

Objective: Until now, giant intracranial aneurysms (GIAs) have in many cases been a vascular disease that was difficult or impossible to treat, not least due to the lack of availability of a large-format stent. In this multicentre study, we report on the first five clinical applications of the Accero^®^-Rex-Stents (Acandis, Pforzheim, Germany) in the successful treatment of fusiform cerebral giant aneurysms. Material and Methods: The Accero^®^-Rex-Stents are self-expanding, braided, fully radiopaque Nitinol stents designed for aneurysm treatment. The stent is available in three different sizes (diameter 7–10 mm, length 30–60 mm) and intended for endovascular implantation in vessels with diameters of 5.5–10 mm. Results: Five patients (all male, age 54.4 ± 8.1 years) with large fusiform aneurysms of the posterior circulation were treated endovascularly using the Accero^®^-Rex-Stents. There were no technical complications. One major ischemic complication occurred. A significant remodeling and reduction in the size of the stent-covered aneurysms was already seen in the short-term post-interventional course. Conclusions: The Accero^®^-Rex-Stents were successfully and safely implanted in all five patients with fusiform giant aneurysms, showing technical feasibility with promising initial results and significant aneurysm size reduction in already available follow-up imaging. Key point: With the Accero-Rex-Stents, a new device is available that offers another treatment option for rare cerebral fusiform giant aneurysms with very large parent vessels.

## 1. Introduction

Giant intracranial aneurysms (GIAs) are cerebral aneurysms with a diameter of at least 25 mm [1,2]. GIAs are thought to develop from a combination of genetic and environmental factors that lead to degeneration of the vessel wall [3,4], resulting in weakening and dilatation of the wall. Risk factors for the development of intracranial aneurysms include hypertension, smoking, positive family history, and connective tissue disease [5]. Elongation and enlargement of the affected artery lead to hemodynamic and hemostatic changes with possible formation of intra-aneurysmal thrombosis, brainstem compression, and increased risk of ischemic complications [5].

The presence of perforating arteries with risk of brain stem infarctions is one of the major challenges facing the endovascular treatment of fusiform aneurysms of the basilar artery (BA) [6]. The overall prevalence of GIAs is higher in women than in men, and they tend to occur more frequently in the anterior circulation than in the posterior circulation [7].

The clinical presentation of GIAs can vary widely depending on the location, size, and associated complications. Common symptoms include headaches, visual disturbances, cranial nerve deficits, seizures, and neurological deficits such as hemiparesis, dysarthria, and cognitive impairment [3,5]. Subarachnoid hemorrhage (SAH) and intracerebral hemorrhage (ICH) are the most serious complications associated with GIAs and are responsible for high morbidity and mortality [2].

Treatment of GIAs is difficult and there is no consensus on the optimal treatment strategy. Traditional treatment options include surgical clipping and endovascular coiling [8,9]. However, these methods have their limitations, especially for large and complex aneurysms. Recently, new endovascular techniques such as flow diversion and stent-assisted coiling have shown promising results in the treatment of GIAs [9,10].

The giant fusiform aneurysms of the BA have specific features compared to aneurysms of the anterior circulation. First, the BA as well as the distal vertebral artery (VA) have to be considered as fully perforator bearing, and second, the diameter of the parent vessel can reach 10 mm or even more, making them untreatable with commonly available intracranial stents.

**Figure 1:**
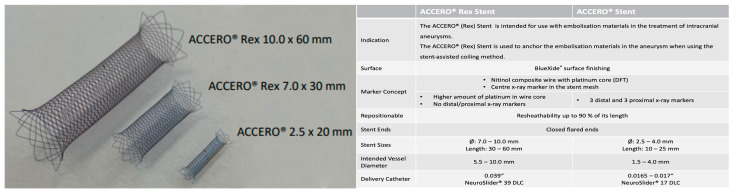
Technical specifications of the Accero^®^-Rex-Stents. The Accero^®^-Rex-Stents are self-expanding, braided, fully radiopaque Nitinol/Platinum (DFT) stents intended for aneurysm treatment. The stent is available in three different sizes (diameter 7–10 mm, length 30–60 mm) and intended for implantation in vessels with diameters of 5.5–10 mm.

The Accero^®^-Rex-Stents are intended for use with embolization materials in the treatment of intracranial aneurysms. They consist of a Nitinol composite wire with a platinum core and a central X-ray marker in the stent mesh. Compared to the established standard Accero^®^-Stents with length between 10–25 mm and a diameter between 2.5–4 mm, they have a higher amount of platinum in the wire core. Despite its size with a length of 30–60 mm and a diameter of 7–10 mm, it possesses the same resheathability. It is intended for the use in vessels with diameters between 5.5–10 mm and can be delivered via 0.039″ NeuroSlider 39^®^ microcatheters (Acandis, Pforzheim, Germany) delivery catheters (see Figure 1).

We report here on the world’s first five cases with overall 12 deployments of the Accero^®^-Rex-Stents in the treatment of fusiform GIAs of the posterior circulation. Our objective is to discuss the technical feasibility of the implantation of these stents in the endovascular treatment of GIA.

## 2. Materials and Methods

In this retrospective, multicentre study, we investigated the interventional treatment of five patients with giant aneurysms of the posterior circulation by using Accero^®^-Rex-Stents between November 2022–August 2023. The patients were treated at three specialized tertiary hospitals with a neurointerventional unit and expertise in aneurysm treatment.

We report on the pre-interventional diagnosis and clinical state of the patients, including clinical and baseline imaging characteristics, as well as technical aspects of the interventional procedures with emphasis on the used material and the feasibility of the procedures in all patients (Table 1). Moreover, we report of the first treated patient in detail as an illustrative case (referred to as patient #1).

### 2.1. Study Population

Inclusion criteria were the presence of giant aneurysms of the posterior circulation and the interventional treatment of these aneurysms with Accero^®^-Rex-Stents. We included n = 5 patients, all male (100%), with a mean age of 54.4 ± 8.1 years. Initial symptoms included diplopia, ataxia, hemiparesis, cephalgia, with gait disturbances being the most prevalent one. All patients showed a worsening of symptoms between initial diagnosis and time of treatment, with the mean time period being 3.8 years.

### 2.2. Image Acquisition

#### Magnetic Resonance Imaging (MRI)

Pre- and postinterventional magnetic resonance imaging (MRI) examinations were performed on a 3 Tesla scanner (Philips Healthcare, Best, The Netherlands) after administration of 0.1 mL/kg of a contrast agent containing gadolinium (Gadovist, Bayer Vital GmbH, Leverkusen, Germany).

MRI protocols included the following sequences: 3D T1 weighted sequences pre- and post-contrast administration, 3D FLAIR sequences, diffusion and susceptibility weighted sequences, and time-of-flight (ToF) sequences.

The MRI images were evaluated by at least two radiologists with at least 6 years of experience in neuroradiological diagnostics.

### 2.3. Computed Tomography (CT), CT-Angiography (CT-A), and CT-Perfusion (CT-P)

Examination was performed with dual source CT (SOMATOM Definition Flash, Siemens Healthineers, Erlangen, Germany, 128 × 0.6 mm) with iodinated non-ionic contrast medium (ULTRAVIST, Bayer Vital GmbH, Leverkusen, Germany) delivered by a power injector after non-enhanced CT of the brain. The examination protocol consists of a non-contrast head CT scan followed by a CT-angiography of the supra-aortic and cerebral vasculature (80 cc contrast medium, 4 cc/s flow rate) while the patients’ arms are lowered. CT-P images were acquired after a delay of 180 s followed by an injection of 30 cc contrast medium at a flow rate of 5 cc/s. Acquisition parameters were 120 kV and 175 mA, the rotation time was 0.5 s, and the pitch was 0.5. Syngovia NeuroPerfusion Software (Siemens Healthineers, Version 5.1) was used for analyzing raw perfusion data. The arterial input function and the venous output function were determined from the middle cerebral artery and the superior sagittal sinus. Maps of relative cerebral-blood-flow (CBF), cerebral-blood-volume (CBV), mean-transit-time (MTT), and time-to-drain (TTD) were calculated automatically by software-based default settings as used in clinical routine.

### 2.4. Neurointerventional Procedure Exemplified by the Intervention in Patient #1

Diagnostic angiography of the cerebral arteries was performed via an arterial transfemoral approach with general anesthesia.

The following materials were used as standard for diagnostic angiography, stent, and flow-diverter implantation in patient #1:

7F Cook sheath (Cook Medical, Bloomington, IN, USA), Sofia EX DAC (Microvention, Saint-Germain-en-Laye, France), NeuroSlider 39^®^ microcatheter (Acandis, Pforzheim, Germany), Terumo standard wire (Radiofocus, Tokyo, Japan), Rebar 18 microcatheter (Medtronic, Irvine, CA, USA, 0.021-inch inner diameter), Traxcess 14 microwire (Microvention, Tustin, CA, USA), Syncro support microwire (Stryker, Salt Lake City, UT, USA), Derivo-Heal-Flow-Diverter 8 × 25 mm (Acandis, Pforzheim, Germany), Eclipse 2L 6 × 20 mm double lumen balloon catheter (Balt, Montmorency, France), and pRESET 6 × 30 mm stent retriever (Phenox, Bochum, Germany).

### 2.5. Drug Therapy during Intervention and after Stent Implantation in Patient #1

#### Pre-interventional drug administration

Clopidogrel 75 mg (1-0-0/d) for five days.

### 2.6. Peri-interventional Drug Administration in Patient #1

An amount of 5000 I.E. Heparin, 500 mg ASA + eptifibatid bolus and infusion (b.w. adapted). Fortecotin: 4 mg (1-1-1/d).

### 2.7. Postinterventional Drug Administration Patient #1

Clopidogrel 75 mg (1-0-0/d) + 100 mg (1-0-0/d) ASA for twelve months. Additionally, 100 mg (1-0-0/d) ASA life-long.

## 3. Results

The patient collective consisted of five patients with large fusiform aneurysms of the posterior circulation. All patients were treated successfully with 2–3 implanted Accero^®^-Rex-Stents of different sizes (Table 1). In all cases, the implantation of the stents was achieved without technical complications. The post-interventional CT or MRI controls showed no/only minor focal ischemia in three out of five cases (patient #2–#4), the neurological status of these patients showed no marked clinical deterioration.

**Figure 2:**
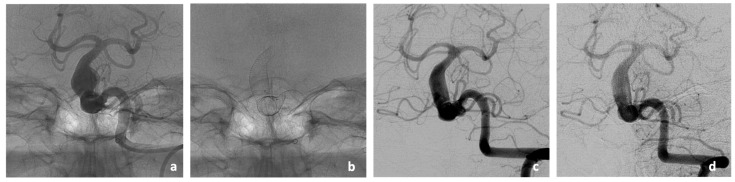
Imaging findings of Patient #2 during endovascular treatment with two Accero^®^-Rex-Stents (**a**,**b**) and three months later (**c**). After implantation of two Accero^®^-Rex-Stents in the BA (**a**,**b**), a clear remodeling of the previously existing aneurysm is already evident in the three-months course (**c**) with stable presentation in the eigth-months follow-up (**d**). Furthermore, there are no signs of in-stent-stenosis or thrombosis.

As an illustrative case of the vessel remodeling effect of the implanted stents, the DSA imaging of patient #2 in the three- and eight-months post-interventional course is given (Figure 2). In this case, the stent-covered aneurysm of the BA showed a significant reduction in size from the previous 11 × 11 mm to only 6 × 6 mm in the follow-up imaging at 3 months and stable size at follow-up imaging at 8 months. The follow-up of patient #5 showed a stable size of the stent-treated aneurysm at 3 months. Two patients died due to pathologies that were not associated with the intervention before follow-up imaging at 3 months could be performed (cardiac co-morbidities in patient #1 and pulmonary sepsis in patient #3). Furthermore, 3 months follow-up of patient #4 was still pending at the time of manuscript submission.

### 3.1. Illustrative Case of the First Patient Treated with the Accero^®^-Rex-Stent

The interventional procedure regarding the endovascular treatment of a fusiform aneurysm of the posterior circulation as well as the postinterventional outcome are described based on the first patient as an illustrative case.

### 3.2. Patient #1

An adult patient presented to the neurological department of our hospital in the year 2018 with known cerebral macroangiopathy and onset of diplopia, gait disturbance, and ataxia. The patient had a pre-existing condition of severe and medicated hypertension, yet the patient was a heavy cigarette smoker (40 pack years).

**Figure 3:**
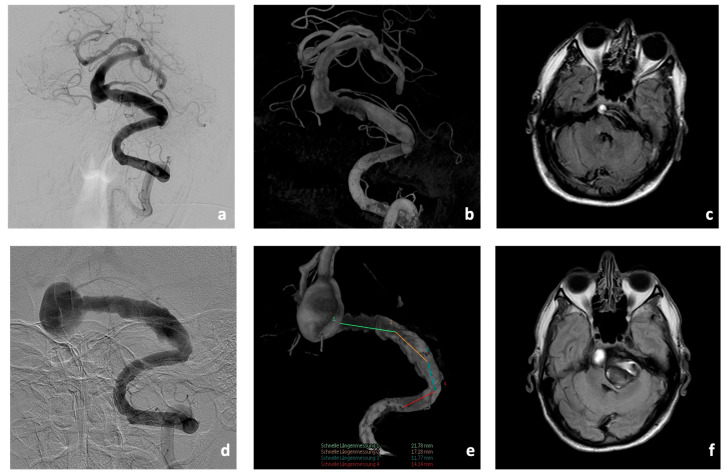
Imaging findings of Patient #1 at initial presentation (**a**–**c**) and four years later (**d**–**f**). Massive progression of the fusiform aneurysm of the V4-segment and the BA in the DSA (**d**,**e**) and in the thrombosed part (**f**) with increasing significant brain stem compression.

Initial cerebral imaging in 2018 with magnetic resonance imaging (MRI), computed tomography (CT), CT-angiography (CT-A), CT-perfusion (CT-P), and digital subtraction angiography (DSA) showed an aneurysm of the BA (10 × 11 mm) with concomitant fusiform caliber irregularities of the V4 segments of the left VA (35 mm in length, Figure 3). In addition, there was a hypoplastic right VA.

At this point, an interdisciplinary consensus was reached between the departments of neurosurgery, neurology, and interventional neuroradiology to initially adopt a wait-and-see approach with optimization of the hypertension medication. Interventional treatment of the giant fusiform aneurysm was deliberately omitted.

Four years later, in 2022, the patient presented to our hospital with a significant worsening of their neurological symptoms with now existing severe dysarthria, dysphagia, hemiparesis of the left side, facial palsy of the left side, and permanent gait disturbance.

CTA showed a significant increase in the size of the fusiform aneurysm with a maximum BA diameter of 15 × 22 mm. In addition, at this time, there was already manifest compression and displacement of the brain stem to the right due to the fusiform aneurysm of the V4 segment of the left VA (8 × 50 mm, Figure 3).

In view of these findings, endovascular therapy of the aneurysm was initiated.

**Figure 4:**
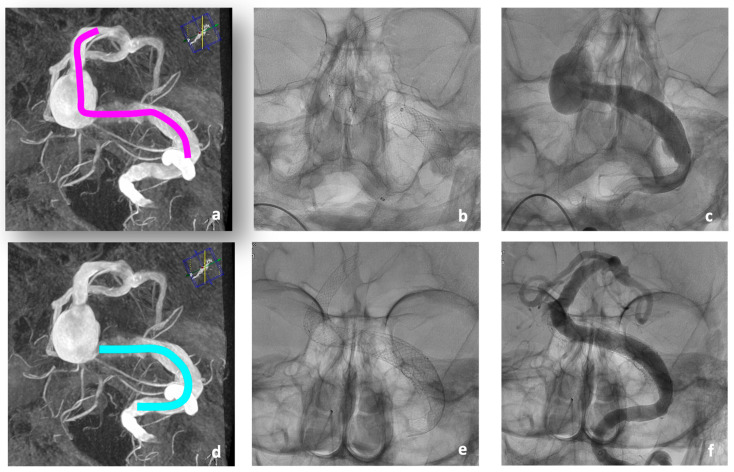
Two-stage treatment of a fusiform aneurysm of the BA and the left V4-segment with a combination of three Accero^®^-Rex-Stents and a Flow-Diverter in situ in patient #1 (**b**,**e**). Schematic drawing of intervention planning ((**a**,**d**), pink: Accero^®^-Rex-Stents, turquoise: Derivo-Heal-Flow-Diverter). In the first intervention, three overlapping Accero^®^-Rex-Stents were implanted (**b**), with no significant angiographic change immediately after implantation (**c**). One week after implantation, there is significant remodeling of the BA and V4-segment (**f**), followed by planned implantation of proximal Flow-Diverter along the thrombosed part (**e**).

After extensive study of a re-performed DSA, a treatment concept was developed that provided for a combined therapy using telescopic braided Accero^®^-Rex-Stents in a first intervention, followed by flow diverter treatment of the aneurysm remnant in a second intervention (Figure 4).

### 3.3. Interventional Procedures

For the first treatment, a Neuron Max^®^ 80 cm 6 F Sheath (Penumbra, Alameda, CA, USA) was directed into the left VA. Coaxially, a Sofia EX intermediate catheter (Microvention, Aliso Viejo, CA, USA) was navigated to the V3 segment. Passage of the aneurysm with a 0.0014” Guidewire was easily possible. Tracking of the deployment catheter, the Neuroslider 39^®^ (Acandis, Pforzheim, Germany), over a wire was only possible up to the proximal BA due to relative rigidity of this large deployment catheter. Instead, the intermediate catheter could be tracked up to the left P1 segment using a tetra-axial approach utilizing a Rebar 18 microcatheter and different guidewires. In this stable position, the Neuroslider 39^®^ could be brought up to the tip of the intermediate catheter, facilitating the deployment of the first and most distal stent. In total, three stents were implanted (one 10 × 60 mm and two 10 × 30 mm) to cover the entire diseased vessel.

No peri-interventional technical complications occurred (Figure 4a–c).

Further, 5 days later, the aneurysm of the BA already showed a partial thrombosis in the post-interventional MRI with regular perfusion of the implanted stents. Several embolic microinfarcts were detected on both sides of the occipital, pontine, and cerebellar region, and the post-interventional neurological status of the patient showed a deterioration with a manifest incomplete tetraparesis.

In the second intervention 7 days later, the treatment was completed by implanting a Derivo-Heal^®^-Flow-Diverter (8 × 25 mm) with coverage of the large aneurysm of the left VA compressing the brain stem (Figure 4d–f). The Accero^®^-Rex-Stents previously implanted in the BA had already led to remodeling of the BA, and extensive thrombosis of the treated BA aneurysm had occurred (Figure 4f). The post-interventional CT showed no new ischemia. The patient showed a persistence of the known tetraparesis.

The patient could be discharged for rehabilitation with antiplatelet therapy of ticagrelor (90/0/90 mg/d) and ASA (100/0/0 mg/d) on the eighth day after the second intervention. Unfortunately, the patient died two months after endovascular intervention due to his cardiovascular co-morbidities.

## 4. Discussion

In this multicentre study, we were able to demonstrate the world’s first and technically successful use of Accero^®^-Rex-Stents in the treatment of five fusiform giant aneurysms of the posterior circulation. Despite the difficult conditions with parent vessel diameters above 7 mm in all cases, the stents were implanted safely, partly in combination with the use of flow-diverters. The first post-interventional MRI or CT scans follow-up showed a remodeling effect with significant reduction in the perfused aneurysm portion and regular perfusion of the inserted stents. The available three-month post-interventional DSA showed a significant reduction in the size of the stent-treated BA aneurysm (patient #2) and a stable size of the stent-treated aneurysm, respectively (patient #5).

Fusiform aneurysms of the cerebral vessels belong to the rarest vascular diseases, representing only 2–3% of all cerebral aneurysms [2]. There are correspondingly few reports of successful treatments available and there is also no consensus on the best therapeutic approach. Therapeutic concepts range from watch-and-wait strategy to endovascular and combined endovascular-surgical approaches [9,11].

With increasing size, aneurysms often show accompanying space-occupying effects with compression of the brain stem as well as smaller infarcts from partially thrombosed aneurysms, which lead to an aggravation of the neurological symptoms [4,5,12,13].

In this context, giant aneurysms of the posterior circulation in particular are very difficult to treat successfully in terms of their outcome and their risk of bleeding; these procedures in particular show a frequent occurrence of peri-interventional complications such as aneurysm rupture [14]. For example, regarding peri-interventional complications, previous studies have shown the occurrence of peri-interventional strokes in up to 23% of treated patients [15].

The treatment of such large aneurysms of the posterior circulation certainly remains challenging and must be questioned very critically regarding its risks [8,10].

Previous limitations in the endovascular treatment options were often the very large diameter of the parent BA or VA. Commercially available braided stents usable and approved for intracranial use have a maximum diameter of 6 mm.

Cases #1 and #2 in our study show the importance of treating such giant aneurysms as early as possible to prevent further growth of the aneurysm, which ultimately increases the complexity and risks of interventional treatment at a later stage [4]. Apparently, it seems particularly important to prevent an aggravation of the neurological symptoms, as the outcome is clearly worse in such courses of disease. In our opinion, it is important to treat a patient as early as possible. Not least because of the very small database, almost every treatment must be considered as an individual healing attempt.

As our cases show, an individual therapy concept for each patient must be developed in an interdisciplinary consensus between the departments of neurology, neurosurgery, and interventional neuroradiology [9]. In particular, the combination of different devices or different techniques must be considered pre-interventional. The results of previous studies regarding aneurysm treatments, for example with the sole use of flow diverters, also showed an improvement in the clinical symptoms for most of the patients, but in individual cases they also led to a significant worsening [16,17].

Our study shows the remodeling effect of the inserted stents over time, which is similar to that of a flow diverter (shown in patient #2 and #5). Unfortunately, current flow diverters are not available in the sizes necessary for the treatment of such large aneurysms. With the availability of new devices such as the Accero^®^-Rex-Stents, the endovascular therapy options available so far can be significantly expanded.

## 5. Technical Aspects

With regard to the technical implementation, we emphasize that the navigation and deployment of such large stents in the posterior circulation requires appropriate access techniques. In two of our cases, the application of an intermediate catheter (Sofia, Microvention or Red 68, Penumbra, Alameda, CA, USA) was useful in guiding the relatively rigid 0.039″ Neuroslider^®^ delivery catheter (Patients #1–#3). The use of two parallel 0.014″ microwires to achieve the deployment position (Patients #4 and #5) is a second feasible technique.

## 6. Limitations

The authors are aware of the limitations of their study, and especially the fact that Accero^®^-Rex-Stents have only been used in five cases in the treatment of fusiform giant aneurysms of the posterior circulation.

Despite the promising preliminary results, the lack of post-interventional follow-up DSA in some patients remains a major limitation to our study.

More frequent use of these stents and further evaluation in larger patient cohorts are needed to confirm our initial positive experience with technical feasibility and promising initial results.

Another minor limitation is formed by the composition of our cohort, which entirely consists of men and GIAs of the posterior circulation, whereas women and the anterior circulation are more often affected according to the literature.

## 7. Conclusions

With the Accero^®^-Rex-Stents, a new device is available that offers another treatment option for rare cerebral fusiform giant aneurysms with very large parent vessels. In this multicentre study, we demonstrate the technical feasibility of the implantation of the stents in patients with GIA. The available follow-up imaging indicate promising results but have yet to be verified by further research.

## Figures and Tables

**Table 1:** Patient and procedural characteristics of five patients treated with Accero^®^-Rex-Stents.

	Patient #1	Patient #2	Patient #3	Patient #4	Patient #5
Comorbidities	dilatative cerebral macroangiopathy, heavy cigarette smoker (100/d), hypertension	hypertension, hepatitis C, history of i.v. drug abuse	urinary tract infection	colitis ulcerosa	colitis ulcerosa, aortic aneurysm, resistance to Plavix
Year of initial diagnosis	2018	2021	2023	2015	2018
Symptoms at initial diagnosis	diplopia, gait disturbance, ataxia	leg-related hemiparesis on the right side	aphasia, hemiparesis on the left side	diplopia, vertigo, ataxia, cephalgies	trigeminal neuralgia
Year of treatment	2022	2023	2023	2023	2023
Symptoms at treatment	severe dysarthria, severe gait disturbance, hemiparesis on the left side, dysphagia	diplopia, dysphagia, constant leg-related hemiparesis on the right side	progredient aphasia, constant hemiparesis on the left side	gait disturbances	trigeminal neuralgia
Endovascular approach	7F long sheath, Sofia EX distal access catheter, Neuroslider 39 Microcatheter, pRESET 6 × 30 mm, Eclipse Ballooncatheter	7F long sheath, RED 68 distal access catheter, Neuroslider 39 Microcatheter, Eclipse Ballooncatheter	6F long sheath, 6F Sofia Plus, Neuroslider 39 Microcatheter	8F guider soft tip, Neuroslider 39 Micocatheter, 2× Choice extra support 0.014″	8F guider soft tip, Neuroslider 39 Microcatheter, 2 × Transend 14 EX
Size and number of used Accero^®^-Rex-Stents	1 × (10 × 60 mm) 2 × (10 × 30 mm)	1 × (7 × 60 mm) 1 × (7 × 30 mm)	2 × (10 × 60 mm)	3 × (8 × 40 mm)	2 × (10 × 60 mm)
Additional implantation of Flow-Diverter etc.	Deriv Heal-Flow-Diverter (8 × 25 mm)	no	no	no	no
Technical complications	no	no	no	no	no
Postinterventional complications	Worsening of neurological status (tetraparesis), MRI: cerebellar and mesencephalic ischemia	no	no	hearing limitations in the right ear	thrombosis of VA covered by the stents after disruption of Prasugrel
3 months follow up	deceased three months after intervention due to cardiac comorbidities	remodeling effect of stent treated aneurysm and reduction of aneurysm size	deceased due to pulmonary sepsis	pending	pending

## Data Availability

The datasets and images used and/or analysed during the current study are available from the corresponding author on reasonable request.
